# Evolution of Regulatory Sequences in 12 *Drosophila* Species

**DOI:** 10.1371/journal.pgen.1000330

**Published:** 2009-01-09

**Authors:** Jaebum Kim, Xin He, Saurabh Sinha

**Affiliations:** Department of Computer Science, University of Illinois at Urbana-Champaign, Urbana, Illinois, United States of America; National Institute of Genetics, Japan

## Abstract

Characterization of the evolutionary constraints acting on *cis*-regulatory sequences is crucial to comparative genomics and provides key insights on the evolution of organismal diversity. We study the relationships among orthologous *cis*-regulatory modules (CRMs) in 12 *Drosophila* species, especially with respect to the evolution of transcription factor binding sites, and report statistical evidence in favor of key evolutionary hypotheses. Binding sites are found to have position-specific substitution rates. However, the selective forces at different positions of a site do not act independently, and the evidence suggests that constraints on sites are often based on their exact binding affinities. Binding site loss is seen to conform to a molecular clock hypothesis. The rate of site loss is transcription factor–specific and depends on the strength of binding and, in some cases, the presence of other binding sites in close proximity. Our analysis is based on a novel computational method for aligning orthologous CRMs on a tree, which rigorously accounts for alignment uncertainties and exploits binding site predictions through a unified probabilistic framework. Finally, we report weak purifying selection on short deletions, providing important clues about overall spatial constraints on CRMs. Our results present a complex picture of regulatory sequence evolution, with substantial plasticity that depends on a number of factors. The insights gained in this study will help us to understand the combinatorial control of gene regulation and how it evolves. They will pave the way for theoretical models that are cognizant of the important determinants of regulatory sequence evolution and will be critical in genome-wide identification of non-coding sequences under purifying or positive selection.

## Author Summary

The spatial–temporal expression pattern of a gene, which is crucial to its function, is controlled by *cis*-regulatory DNA sequences. Forming the basic units of regulatory sequences are transcription factor binding sites, often organized into larger modules that determine gene expression in response to combinatorial environmental signals. Understanding the conservation and change of regulatory sequences is critical to our knowledge of the unity as well as diversity of animal development and phenotypes. In this paper, we study the evolution of sequences involved in the regulation of body patterning in the *Drosophila* embryo. We find that mutations of nucleotides within a binding site are constrained by evolutionary forces to preserve the site's binding affinity to the cognate transcription factor. Functional binding sites are frequently destroyed during evolution and the rate of loss across evolutionary spans is roughly constant. We also find that the evolutionary fate of a site strongly depends on its context; a pair of interacting sites are more likely to survive mutational forces than isolated sites. Together, these findings provide new insights and pose new challenges to our understanding of *cis*-regulatory sequences and their evolution.

## Introduction

Gene regulation is well recognized as a major determinant of how an organism functions [1], and is also gaining recognition as an important evolutionary substrate [2],[3]. Transcription control is one of the most common forms of gene regulation, and is known to be implemented through regulatory sequences often in the neighborhood of genes. Binding of transcription factors (TFs) to certain positions within regulatory sequences enhances or inhibits transcription and these bound sequences are called transcription factor binding sites (TFBSs). In the case that a gene has to be combinatorially regulated by multiple transcription factors, the cognate TFBSs of those regulating factors tend to be clustered together in ∼1 Kbp-length sequences called “*cis*-regulatory modules” (CRMs), or simply “modules” [4].

Despite significant recent efforts [5]–[8], we lack a good understanding of the organizational principles of CRMs, e.g., the requirements on strengths and arrangements of binding sites within a particular CRM. Inter-species comparison of modules provides a major opportunity to improve our understanding of such principles: (i) Evolution of CRM sequences is constrained by functional requirements, so the study of CRM evolution should allow us to infer which underlying features are more important, and to what extent. (ii) One may hope to find certain evolutionary signatures of CRM sequences through careful inter-species analysis [9], greatly facilitating the identification of yet unknown CRMs. (iii) The study of CRM evolution will also enable us to better understand the path “from DNA to diversity” [10].

Transcription factor binding sites are commonly predicted based on the assumption of their evolutionary conservation [11]. However, the exact nature of their conservation presents a complex picture. The study by Moses et al. [12] in yeast revealed that the rates of change of nucleotides of a TFBS depend on the binding profile of that TF–the positions of more specific protein-DNA binding permit lower rate of change. It should therefore be possible to leverage the position-specific substitution pattern to better predict TFBSs, as was done in [13]. This pattern has also been reported in bacteria [14] and vertebrates [15], but not in *Drosophila*. Given that this evolutionary pattern has already been assumed in practical analysis [16], it seems worthwhile to verify it in *Drosophila*. Moses et al. [13] further assumed that evolution of nucleotides at different positions are independent, and existing models of binding site evolution [17],[18] rely on this assumption; however, its validity is not obvious, given that a binding site typically functions as a unit. Empirical evidence either for or against this assumption has been lacking, except for a study in bacterial evolution [19] (where the evidence was against it). There is thus a clear need to test existing and new models of binding site evolution on the multi-species data from different phyla.

Even the most fundamental assumption of regulatory comparative genomics, that binding sites are evolutionarily conserved, has been challenged–Emberly et al. [20] found that binding sites are not substantially more conserved than their adjacent sequences in *Drosophila*; also, TFBSs are often found to have an unexpected amount of flux (gain or loss) in known CRM sequences [21]–[23] and in TF-bound regions in *in vivo* binding assays [24],[25]. It has been suggested that this flux is in part due to expression changes in the genes controlled by these sequences [24], and in part due to weak selection on individual sites even if the expression pattern of the target gene is conserved [26]. However, quantitative estimation of the strength of selection on binding sites has rarely been made, and requires extensive data on sets of orthologous binding sites. Moreover, the question of what leads to the observed levels of TFBS loss and gain is far from being resolved. For example, are the sites with higher binding affinities more likely to be conserved in evolution? How does the local context, i.e., the presence of other sites in the neighborhood, affect the probability of loss of a site? Does the loss probability correlate with overall selective pressure (substitution rate) of the CRM?

Cameron et al. [27] showed that insertions or deletions (“indels”) may be a powerful predictor of CRM sequences in sea urchin, as long indels were suppressed inside CRMs relative to their neighboring sequences. Lunter et al. [28] speculated that such a selection pattern may be particularly relevant to CRMs, as the “fitness” of these sequences may be sensitive to the *length* of the sequences between adjacent TFBSs, but not their exact nucleotide composition. In several earlier studies involving a number of well-studied CRMs in *Drosophila*, such a pattern has not been fully observed [23],[29]. So the following question remains: is indel-purifying selection in regulatory sequences a general evolutionary force, common to different organisms? The answer will affect our understanding of CRM organization; e.g., how tolerant a CRM sequence is to the change of spacing between TFBSs.

Earlier attempts to characterize the evolutionary patterns of regulatory sequences used a few well-studied CRM sequences. These studies were limited in their scope [21],[23],[29]. The availability of 12 *Drosophila* species [30] and a large collection of experimentally verified *Drosophila* CRM sequences [31] enable a large-scale and more systematic study of the evolutionary patterns of CRM sequences. Such studies also crucially depend on accurate computational tools for sequence comparison. Commonly used multiple alignment tools [32]–[34] that treat regulatory sequences as no different from other types of DNA (or for that matter amino acid) sequences are known to be a source of errors in evolutionary analysis [35],[36]. Even if the alignments are accurate, the step of annotating gaps as insertions or deletions (usually done by *ad hoc* parsimony criteria) may lead to inaccurate inferences [37]. We have previously developed new methods for inter-species sequence analysis, that are specially designed with the properties of regulatory sequences in mind. These include (i) Morph [38], which optimizes pair-wise sequence alignment by using the known binding profiles of relevant transcription factors, and (ii) Indelign [39], which uses a realistic probabilistic model of insertions and deletions to annotate “indel” events in a given multiple alignment. In this work, we take advantage of and extend these new methods to study the CRMs involved in *Drosophila* early development. This data set is ideally suited for such research because (i) the biological system is very well studied [8] and the relevant transcription factors are known, thereby limiting the false positives in binding site annotation, and (ii) much of the previous work on metazoan *cis*-regulatory evolution has been in this system [7],[23],[26]. Our study significantly extends the earlier work done on this dataset [40] and provides answers to many of the burning questions alluded to above.

## Results

### TFBS-Conscious Multiple Alignment and Binding Site Annotation

We begin with our findings on the evolutionary behavior of transcription factor binding sites. We collected 68 *D. melanogaster* CRMs and seven TF motifs involved in the control of anterior-posterior segmentation in the blastoderm stage embryo. These CRMs (source: REDfly [31]) have been experimentally determined, without using evolutionary conservation for discovery, and are hence suitable for evolutionary studies without introducing ascertainment bias. Orthologous sequences of these CRMs were extracted from 11 other *Drosophila* species and were aligned by a special multiple alignment program, called “ProbconsMorph”. This is a new computational tool that we have developed, and is geared towards multiple alignments of regulatory modules in a TFBS-conscious manner (see Methods). It avoids propagating pair-wise alignment errors to the entire multiple alignment by combining the “consistency transformation” (see Methods) of Probcons [41] with posterior alignment probabilities obtained from Morph [38]. We also repeated most of our tests using the alignment tool “Pecan” [42] that does not use TF motifs, and we point out differences, if any, between results from the two types of alignment.

We annotated binding sites for each transcription factor, in the subset of *D. melanogaster* CRMs that overlap with ChIP-bound regions from Li et al. [43], if such data was available. Site prediction was based on the p-value of match to the respective PWM (“position weight matrix”) motif. We contrasted the density of these binding site predictions (in “bound” CRMs) with those in “unbound” intronic sequences, and typically found 2–3 fold enrichment in the former. (See Text S1, “False positive proportion estimation”.) We also predicted sites in each of the 11 other species separately, using the same method. Considering a binding site to be conserved if it is present in all other species in the *D. melanogaster* subgroup, we found that conserved sites were 2–3 fold enriched in CRMs than in intronic sequences. (See Text S1, “False positive proportion estimation”.) Our findings are consistent with earlier results in Li et al. [43], suggesting that the majority of predicted sites are likely to be functional.

Binding sites from different species, that overlap each other in the multiple alignment, are collectively referred to as an “orthologous TFBS set”. Sites in such an orthologous set were re-aligned locally in order to correct for any errors in their precise alignment. Graphic visualizations (Figure S1) of these 12-species CRM alignments, with binding site annotation, are available at our site http://europa.cs.uiuc.edu/TFBSevolution/.

### Binding Sites Have Position-Specific Substitution Rates

**Figure 1:**
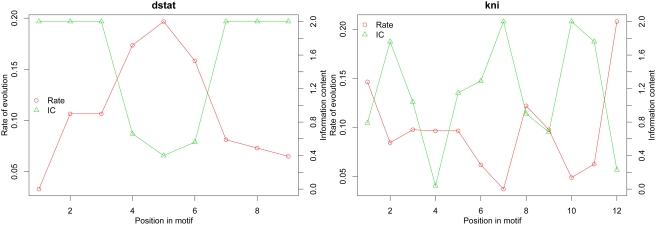
Correlation between the specificity of a TFBS position and its evolutionary rate in transcription factors *Dstat* and *Kni*, with Pecan alignments.

Different positions in binding sites have different contributions to the binding affinity of the TF. Positions that form the core regions for TF-DNA binding are more specific (less variation allowed) in the motif, and should be under stronger selective constraints. We thus expect different positions of TFBSs to have different degrees of evolutionary conservation. The specificity of a position can be expressed by the information content (IC) of the corresponding column in the PWM (position weight matrix), and the evolutionary rate by the number of substitutions in that position in orthologous binding sites (see Methods). We observed highly significant negative correlations between specificity and evolutionary rate in five of seven TFs (i.e., all except *Cad* and *Tll*) (Table 1; Figure 1; Figure S2). Thus, our results confirm earlier similar findings in bacteria, yeast and vertebrates [12],[14],[15]. To avoid a bias introduced by the use of PWM-guided alignments, we used Pecan alignments (see Methods) of five closely related species for this particular analysis. The results were reproduced when using ProbconsMorph alignments (Table S1; Figure S3).

**Table 1:** Correlation between the specificity of a TFBS position and its evolutionary rate, with Pecan alignments.

Factor	Number of TFBSs	Width of motif	Correlation coefficient^a^	P-value
bcd	160	8	−0.75	**0.0153**
cad	175	9	−0.48	0.0969
dstat	129	9	−0.83	**0.0031**
hb	170	8	−0.69	**0.0347**
kni	85	12	−0.82	**0.0005**
kr	177	11	−0.53	**0.0457**
tll	185	10	−0.38	0.1375

a Spearman's correlation coefficient.

### Selection Acts on Entire Binding Sites

While substitution rates in a TFBS are position-specific, this does not imply that different positions evolve independently, although such an assumption is often made in existing evolutionary models [17],[18],[44]. It is easy to see that the exact same substitution can have drastically different effects on the functionality of a site, depending on how strong the site was to begin with. A site that is close to optimal will probably remain a site even if a crucial nucleotide is changed, thus this substitution is likely to be fixed. On the other hand, the same nucleotide change inside a weak site may have a larger functional consequence (the site loses its binding functionality), thus will be less likely to be fixed. It therefore seems plausible that the substitution rate of a position should depend on the entire site.

To study evolution at the level of binding sites, as opposed to nucleotides, we developed a simple mathematical model of binding site evolution, called “Site-level Selection” or “SS” model, that treats binding sites as single evolutionary units. Under this population genetics-based model, the fitness of a site can take two values, 1 if the binding affinity of this site is below some threshold, and 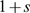 if the affinity is above this threshold, for 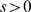. (We use the same threshold as that used for defining a binding site.) The rate of substitution from site 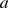 to 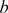, 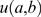, is determined by the fitness difference between 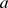 and 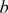 according to this equation from population genetics theory [19],[45]:
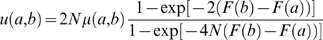
(1)where 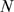 is effective population size, 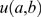 is the mutation rate of 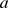 to 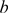, and 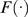 is the fitness function defined above. When 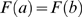, we have 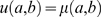; when 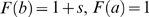, i.e., there is a site gain, we apply the approximation that 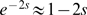:
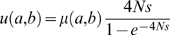
(2)


When 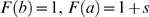, i.e., there is a site loss, similarly we have:
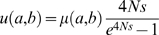
(3)


Note that 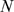 and 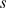 are inseparable in the above equations, so we will use the single quantity 4*Ns* as measuring the intensity of selection.

**Figure 2:**
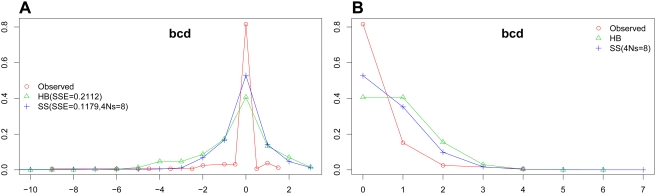
Distributions of evolutionary changes in observed binding sites (Observed), and those simulated by Halpern-Bruno (HB) and Site-level Selection (SS) models for the transcription factor *Bcd* in *D. melanogaster* and *D. yakuba* species pair. (A) Distribution of energy difference between a predicted binding site in *D. melanogaster* and its orthologous site in *D. yakuba*. The x and y axes represent energy difference and frequency respectively. (B) Distribution of the number of substitutions between *D. melanogaster* and *D. yakuba* sites. The x and y axes represent the number of substitutions and frequency respectively. SSE denotes the sum of squared errors between the observed and the simulation-based distributions and “*4Ns*” denotes the optimal value of this free parameter of the SS model.

We tested how well this model fits the data on binding site evolution, and compared it to another model, called the “Halpern-Bruno” or “HB” model [17], which assumes positional independence and purifying selection at each position of the TFBS. The HB model has been used previously in *cis*-regulatory analyses (e.g., Moses et al. [13]). We considered predicted binding sites in *D. melanogaster* and their respective aligned sequences (whether designated binding site or not) in a closely-related species (*D. yakuba*), arbitrarily calling the former sites “ancestral” and the latter sites “descendant”. Assigning an “energy score” to each binding site based on its similarity to the PWM [46], we calculated the difference in energy score between the ancestral and descendant sites, and used this as the statistic to represent binding site evolution. We computed, for each TF, the histogram of this “energy difference” statistic, and asked how well this histogram fits theoretical predictions from simulations using either the SS or the HB model (Table 2). For every motif, the SS model showed a significantly better fit to the data than the HB model. (Table 2; Figure 2A; Figure S4). (See Methods for details of how statistical significance was estimated, while accounting for the additional free parameter in the SS model. The results were reproduced when using Pecan alignments; see Table S2 and Figure S5.) Our estimated level of selection (4*Ns* in the range 8–19) is consistent with an early estimate from bacterial regulatory sequences [19] and our results argue in favor of models treating entire binding sites as evolutionary units.

**Table 2:** Comparison of HB and SS models.

Factor	Median SSE^a^		P-value^b^	*4Ns* c
	HB model	SS model		
bcd	0.19	0.10	<2.20E-16	8
cad	0.23	0.16	<2.20E-16	8
dstat	0.12	0.06	<2.20E-16	11
hb	0.10	0.07	<2.20E-16	15
kni	0.21	0.15	<2.20E-16	19
kr	0.19	0.15	<2.20E-16	8
tll	0.18	0.10	<2.20E-16	17

a Median values of sum of squared errors (SSE) from 100 different simulations with the model.

b P-value from paired Wilcoxon signed-rank test.

c Optimal value of the free parameter of SS model.

However, in absolute terms, neither model explains the data very well (Figure 2A; Figure S4), and there is a greater amount of conservation (energy differences close to zero) in the observed data than predicted even with strong selection. A similar analysis was performed with the evolutionary statistic being the number of substitutions between ancestral and descendant sites, and we found that there is an excessive number of fully conserved sites (no substitutions) than expected under either the HB or the SS model (Binomial test, p-value<10^−12^) (Figure 2B; Figure S6; Figure S7 with Pecan alignments). This seems to indicate that for many sites, the allowed binding affinities fall in some narrower range, instead of being determined by a single threshold (lower bound). It has been suggested that in order to produce the correct expression pattern, a binding site may prefer some specific affinity level, and both stronger and weaker binding tend to be less functionally optimal [47]. Our results provide support for this hypothesis.

### TFBS Turnover Follows a Molecular Clock

**Figure 3:**
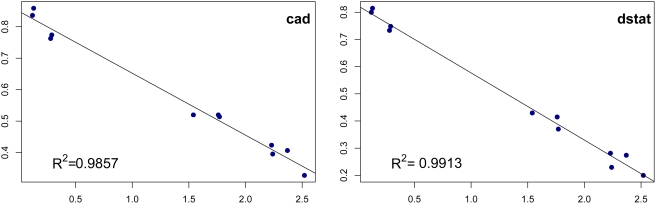
The fraction of *D. melanogaster* TFBSs that are conserved in a related species (y-axis), as a function of the divergence time to that species (x-axis), for transcription factors *Cad* and *Dstat*.

Even though TFBS loss and gain (henceforth called “turnover”) have been commonly observed, it is not clear whether these changes are adaptive [48] or not [26]. If adaptive selection is the main force behind binding site turnover, it is likely that the process will show a lineage-specific pattern; on the other hand, a molecular clock has been known to be suggestive of the absence of adaptive selection, as per the neutral theory of evolution [49]. We considered the fraction of binding sites in *D. melanogaster* that have an ortholog (above threshold) in a second species, and plotted this fraction as a function of evolutionary divergence from the second species (Figure 3; Figure S8). For all transcription factors, the fraction of shared binding sites decreases linearly (*R^2^*>0.90, Table 3) as the divergence time increases, a clear sign of a molecular clock. One problem that may confound the analysis is the presence of false positive binding sites predictions, which are expected to follow a molecular clock. To examine this effect, we calculated a correction term in the fraction of conserved sites, and regressed this with divergence time, using the false positive proportion as a free parameter. High values of the adjusted *R^2^* were obtained (Table 3), confirming the presence of the molecular clock. We repeated the exercise with sites for randomly created PWMs, and found a similar linear relationship. The rate of loss (negative slope of the line) for these random sites is higher than the rates for binding sites, for six of the seven transcription factors (Table 4), the difference being significant for *Bcd* and *Kr*. We note that the sites predicted by random PWMs do not represent neutral sequences, but reflect the average constraint in CRM sequences. This has been shown previously in [43]. The results were reproduced when using Pecan alignments (Table S3 and S4; Figure S9).

**Table 3:** Goodness-of-fit of a linear model for the fraction of conserved binding sites over divergence time.

Factor	R^2^ (raw data)^a^	Adjusted R^2^ (corrected data)^b^	FP^c^
bcd	0.9813	0.9631	0.14
cad	0.9857	0.9693	0.29
dstat	0.9913	0.9831	0.26
hb	0.9114	0.9180	0.24
kni	0.9642	0.9883	0.31
kr	0.9698	0.9097	0.27
tll	0.9894	0.9515	0.32

a R^2^ from raw data without correcting for the false positive rate.

b Adjusted R^2^ from data corrected for the false positive rate.

c Estimated false positive rate obtained by regression.

**Table 4:** Comparison of loss rates of binding sites using real and random motifs.

		Random PWMs	
Factor	Loss rate	Mean	Stdev
bcd	0.1865	0.2530	0.0217
cad	0.1969	0.2444	0.0213
dstat	0.2471	0.2642	0.0172
hb	0.1470	0.1937	0.0211
kni	0.2315	0.2551	0.0170
kr	0.1811	0.2666	0.0172
tll	0.2147	0.2389	0.0191

These rates are without false positive correction.

### Evolution of TFBSs Is Affected by Binding Site Strength and Local Context

Having characterized some general patterns of TFBS evolution, in this section we study what specific factors may influence the conservation and turnover of binding sites.

#### Binding site strength

We defined the strength of a site as the degree of match of this site to the corresponding motif, as measured by a log-likelihood ratio (*LLR*) score ([46], also see Methods). TFBS turnover was defined as the number of TFBS losses in unit evolutionary time (see Methods for the exact definition). We focused on TFBS losses to avoid a possible ascertainment bias due to spurious binding site annotations when analyzing binding site gain events. We observed significant negative correlations between TFBS strength and turnover, for six TFs (i.e., all but *Cad*) (Table 5; also see Methods for details of test). We note that our alignment procedure, which uses motifs to construct and adjust the alignment, will tend to put strong sites in aligned positions and thus make them seem more conserved. We tested for such a bias by repeating the exercise with random PWMs that preserve the information content and G/C content of the original motifs. For each of the six motifs with significant correlations, their 100 randomized versions almost always had less significant correlations (see Table 5). The results were reproduced when using Pecan alignments (Table S5).

**Table 5:** Correlation between TFBS strength and TFBS turnover rate.

Factor	Number of TFBS sets	Correlation coefficient^a^	P-value	Random PWM^b^
bcd	163	−0.71	**0.0002**	0
cad	168	−0.30	0.0974	18
dstat	129	−0.46	**0.0221**	11
hb	168	−0.62	**0.0030**	0
kni	86	−0.58	**0.0025**	4
kr	191	−0.72	**0.0002**	0
tll	188	−0.86	**<2.20E-16**	0

a Spearman's correlation coefficient.

b Number of random PWMs (out of 100 simulations) that show greater correlation than the real motif.

#### Constraint on CRM

Another potential determinant of turnover is the overall evolutionary constraint on the CRM to which the site belongs. We estimated the substitution rate for each CRM using the Paml software [50] and correlated it with the overall TFBS turnover rate of that CRM (see Methods). We found a significant correlation only for one of the seven factors (*Dstat*, p-value 0.0127, Table S8), but this finding was not confirmed by the Pecan-based alignments (Table S9).

#### Homotypic clustering

A “homotypic TFBS cluster” [51] is a group of binding sites of the same TF, often found in the enhancers controlling early development in *Drosophila*. A homotypic TFBS cluster is thought to impart redundancy to the *cis*-regulatory apparatus, and should exhibit greater tolerance towards the loss of sites as compared to a CRM that has only one or two binding sites of the same factor. To test this, we computed the degree of homotypic clustering in a CRM as the number of putative *D. melanogaster* sites in the sequence (normalized by the sequence length), and correlated it to the overall TFBS turnover rate of the CRM (see Methods). However, no significant correlation was observed (data not shown).

We next examined spatial proximity of homotypic binding sites at a finer granularity: if two adjacent sites of the same factor are closely located, there may be cooperative binding of the factor to these sites, leading to stronger selective pressure. Such cooperative binding by proximal sites is known for the *Bcd* transcription factor [52]. We calculated, for each TFBS, the distance to the closest site of the same factor and the distance was correlated with the turnover rate (Table 6). We found significant positive correlations for the factors *Cad*, *Hb* and *Tll*. The only difference from Pecan-based alignments (Table S6) is the positive but weaker correlation for *Cad* (p-value 0.15), perhaps due to misalignments (see Discussion). Surprisingly, *Bcd* did not exhibit any significant correlation in our tests with both types of alignments.

**Table 6:** Correlation between the distance between two adjacent homotypic sites and TFBS turnover rate.

Factor	Number of TFBSs	Correlation coefficient^a^	P-value
bcd	157	0.04	0.3969
cad	162	0.38	**0.0184**
dstat	112	0.00	0.5000
hb	156	0.30	**0.0406**
kni	82	0.24	0.1270
kr	183	0.14	0.2212
tll	178	0.30	**0.0479**

a Spearman's correlation coefficient.

#### Proximity or overlap of heterotypic sites

Binding sites often “interact” with sites of other factors in their neighborhood. Such interactions may include, for example, cooperative binding to DNA or short-range repression. We next examined the effect of spatial context of “heterotypic” binding sites on evolutionary constraint. In a procedure similar to that of Hare et al. [53], we classified sites as belonging to the “proximal”, “distal” or “overlap” class depending on whether the closest site of another factor was within 10 bp, more than 10 bp away, or overlapping. We found sites in the “overlap” or “proximal” categories to be more conserved (present in all 12 species) as opposed to sites in the “distal” category (p-value 4.39×10^−5^ in ProbconsMorph based alignments, p-value 0.001 in Pecan alignments, Hypergeometric test).

We next tested the effect of the above spatial categories individually for each factor. Comparing the “proximal” and “distal” classes (Table 7, column “P vs D”), we found *Dstat* and *Tll* sites to be significantly more conserved when having a proximal partner site (p-value 0.021 and 0.027 respectively). The same results were found using Pecan alignment (Table S7). Interestingly, *Bcd* sites had a significant (p-value 0.012) tendency to be non-conserved (i.e., not present in all 12 species) if they had a proximal partner (Table 7).

**Table 7:** Binding site conservation and its spatial context.

Factor	P vs D^a^	O vs NO^b^
bcd	0.9981*	0.5910
cad	0.5626	**0.0015**
dstat	**0.0213**	0.8981
hb	0.2141	**0.0174**
kni	0.4784	0.2425
kr	0.2071	**0.0387**
tll	**0.0275**	0.0806

Numbers are P-values from hypergeometric test.

a P means proximal and D means distal.

b O means overlap and NO means non-overlap.

*: The opposite p-value is 0.0124.

In a similar comparison of the “overlap” class with its complement (“proximal or “distal”) (Table 7, column “O vs NO”), *Cad*, *Hb*, and *Kr* sites showed a tendency to be more conserved when having an overlapping partner (p-values 0.002, 0.017, and 0.039 respectively). These results were reproduced when using Pecan alignments (Table S7).

In summary, five of the seven motifs showed a significant tendency to be conserved when they had a partner either overlapping with or proximal to them. *Kni* was the only motif examined without such a property, and it is worth noting that *Kni* has the fewest sites in our collection. We also repeated the above test with the requirement that the “partner” site be that of a repressor (*Kni*, *Kr*) when studying an activator TF (*Bcd*, *Cad*, *Dstat*) and vice versa. We found a significant result only for one TF (*Cad*, p-value <0.05), and not for other factors, potentially due to small sample sizes (data not shown).

### Deletions but Not Insertions Are Significantly Underrepresented in CRMs

Finally, we analyzed insertions and deletions in known regulatory sequences, to study the extent of indel-purifying selection. Among 370 non-overlapping *D. melanogaster* CRMs from the REDfly database [31], we chose 128 CRMs that have clear orthologous sequences in *D. simulans*, *D. yakuba*, and *D. erecta*. This choice of species was dictated by simulation-based assessment of the limits of our indel annotation capability (see Methods). Because insertions and deletions (indels) may have different functional consequences on CRMs, we treat them differently. We estimated the number of short insertions and deletions in CRMs using Pecan [42] for alignment and Indelign [39] to annotate the indels. For each CRM, the insertion or deletion count was defined as the average of the respective counts in the four species, weighted by the branch length. We compared indel frequencies in CRMs to those in “background sequences”, chosen to be the regions flanking the CRMs. We found that (i) the number of short deletions (less than 20 bp in length) in CRMs is significantly smaller than that in background regions (paired Wilcoxon signed-rank test, p-value 0.0074; 1970 in CRMs and 2183 in length-matched background regions) and (ii) there was no statistically significant difference (p-value 0.5464) in the number of insertions (1932 in CRMs and 1870 in background). The number of long indel events (20 bp or longer) in our data set was relatively small (CRM: 107 insertions and 175 deletions, background: 115 insertions and 178 deletions) and no significant difference was observed in this regard between CRMs and background regions.

Another related question is the indel pattern in the “spacer” region between CRMs and transcription start site (TSS) of the target genes. Transcriptional regulation depends on the communication between CRMs and promoter sequences [54], which may pose some requirements on the length of the spacer sequences. We thus repeated the above analysis on these spacer regions. (We only consider 63 upstream CRMs in this experiment.) No significant differences in frequencies of insertions or deletions were observed between these regions and background sequences (data not shown).

Our results show that indel-purifying selection exists on CRM sequences, but such selection acts most strongly on deletions. We did not find clear suppression of long-indels, as has been observed before [27].

## Discussion

The study of *cis*-regulatory evolutionary patterns has provided important insights on regulatory sequence function [23],[55], and proves valuable for prediction of these sequences in genomes [9],[56]. Yet, our understanding of *cis*-regulatory evolution is limited at best. While we have theories as well as a large volume of empirical data on protein evolution, we essentially have no theory and have made limited observations on the evolution of regulatory sequences. Our goal here is to begin to bridge the gulf between the vast amount of genomic sequence data and our poor understanding of regulatory sequences and their evolution. We have conducted a detailed evolutionary analysis of a large collection of experimentally verified CRM sequences, taking advantage of the recently sequenced 12 *Drosophila* genomes. Our analysis has revealed several interesting patterns, some along expected lines (but not confirmed previously), and some contrary to our expectations. We believe that our work will furnish evidence orthogonal to experimental characterization for understanding the organizational principles of CRMs, and will be important for developing a theory of regulatory evolution in the future.

There are several technical issues that were important to address in our analysis. Evolutionary comparison depends on the alignment of orthologous sequences, but in general, alignments cannot be perfectly determined and may be a source of biased conclusion [36]. This may be a particularly serious problem for the analysis using 12 *Drosophila* species because of the relatively large divergence. We addressed this concern by developing a new multiple alignment program tailor-made for regulatory sequences. It combines the power of a pair-wise regulatory sequence alignment tool, Morph [38], and a probabilistic multiple alignment framework Probcons [41]. We have made this new software (ProbconsMorph) available freely for public use, to facilitate future studies of this genre. Nevertheless, the use of motifs to construct alignment may artificially boost the conservation level of TFBSs. We carefully addressed this potential bias whenever it may affect our conclusion. For example, when testing the positional variation of substitution rates, we use Pecan-based alignments without using motifs and limited ourselves to five closely related species. Similarly, when testing the correlation of binding site strength to turnover rates, we use randomized PWMs (as “negative controls”) to validate our finding. We also repeated all our analyses with Pecan-based alignments. The various trends seen in Results were almost always reproduced. One notable difference was that the correlation between nearest homotypic site distance and evolutionary rate (Table 6) for *Cad* was statistically significant (p-value 0.02) in ProbconsMorph alignments, but insignificant (p-value 0.15) in Pecan alignments. We suspect that this may be due to the tendency of standard alignment tools (such as Pecan) to misalign one or two nucleotides at the boundary of binding sites, especially if the motif contains short repeats such as TTTT [38], as is the case for *Cad*.

Another critical component of our analysis is the prediction of TFBSs. By using the same PWMs for all the genomes, we have made the assumption that the PWM of any TF is fully conserved across 12 *Drosophila* genomes. This is questionable, as researchers have found in yeast that the change of TF binding specificities can be an important part of the evolutionary change of regulatory networks [57]. For the seven motifs we analyzed, however, there is prior computational evidence that the binding specificities have not changed between *D. melanogaster* and *D. pseudoobscura* [58]. Another issue related to TFBS prediction is that predicted binding sites tend to have a high proportion of false positives [59]. We believe this problem is mitigated by our focus on the segmentation network, the fact that we restrict ourselves to transcription factors and CRMs experimentally known to be involved in regulating the segmentation genes, and our use of ChIP-based binding information wherever possible. We also believe that within a CRM, any computationally predicted binding site for a relevant transcription factor can “attract” transcription factor molecules, and contribute to the expression pattern, and should thus be considered “functional” in a broad sense. The results from Janssens et al. [60] seem to support this point. In practice, we may still have a small number of false predictions because of inaccuracies of the PWMs and we have attempted to estimate the false positive proportion by various methods (see Text S1). Also note that while false site predictions may obscure the evolutionary pattern of functional binding sites, they will not, in general, introduce spurious patterns (since, by definition, these sites are not under selection). In cases where the false sites may affect our interpretation of results, for example, in the test of molecular clock for binding site turnover, we have tried to make appropriate corrections. In addition, in estimating TFBS turnover rates, we have emphasized on losses rather than gains of sites, because a predicted TFBS loss event has stronger supporting evidence than a gain event (the “gained” site is more likely to be a false positive prediction).

Our model of binding site evolution, the “Site-level Selection” (SS) model, is a special case of the population genetic model proposed by Mustonen and Lassig [19]. Under their model, the fitness of a site is determined by its binding energy. The difference of the energy distribution of known sites and of the neutral sites allows one to estimate the fitness of any energy value. A binding site evolves in the space of all possible sequences, with the transition rate between any two sequences determined by the fitness values of the two sequences, given by Equation (1). For most known TFs, however, the number of known sites is too small to reliably estimate a fitness function and the simplification introduced in our model is probably necessary. Our SS model is also similar to the model in Raijman et al [61]. Under this model, a site always tends to preserve its current functional status, that is, the substitution in a binding site that makes is nonfunctional will have a lower rate, and similarly, a substitution that creates a functional site in an originally neutral site will also have a lower rate. However, their model is not formulated in population genetic terms and the transition from a non-site to site is always selected against (this will be favored under the Mustonen-Lassig model and ours). We found that the SS model better explains the evolutionary pattern of binding sites than the HB model, which assumes the independence of substitutions at different positions of a site. A recent study [62] also reported this dependence of binding site positions, though without directly comparing two kinds of models. Admittedly, the presence of false sites may complicate our analysis. It is difficult to directly address this issue, say, through a mixture model approach as done in [22] because of the difficulty of computing probabilities under the SS model. However, we note that if we were to remove false sites from the observed data, we would see a greater proportion of conserved sites, implying that the SS model will continue to be closer to the observation than the HB model (see Figure 2). Next, we observe an overrepresentation of fully conserved sites (no mutations) compared to what is expected from both SS and HB models (Figure 2B). This argues for the conservation of precise affinities, a hypothesis consistent with our current knowledge about the dependence of expression pattern on precise binding affinities [63],[64], though this phenomenon has not been statistically observed previously. Finally, we note that the findings of position-specific substitution rates and site-level selection are not contradictory; as pointed out in [19], each position of the site contributes separately to the fitness of the site, which depends on the sum-total of these contributions.

Our findings of a molecular clock extend earlier results on a small number of well characterized CRMs [65] across three *Drosophila* species, suggesting that this is a property common to developmental CRMs across a large evolutionary range. Even though we cannot exclude the presence of adaptive selection in individual cases, our results seem to suggest that negative selection to maintain the existing binding sites is the dominant mode of evolution, coupled with the occasional loss of sites due to random drift. The rate of site loss likely reflects the strength of purifying selection.

Our tests point out that stronger binding sites are conserved more often than weaker sites. This is consistent with an earlier study [66], which found that stronger *Dorsal* binding sites were more likely to reside in conserved blocks. A simple explanation for this is that stronger sites are more likely to be important to CRM function, thus under stronger constraint. An alternative explanation is that there is a “quality” threshold that defines functionality and once a site drops below that threshold, it is impervious to selective forces. Assuming this is true, we note that a weaker site is closer to the threshold than a stronger site, and may thus be lost more easily. A recent paper [61] seems to support the latter hypothesis. It is likely that the forces of natural selection as well as those of mutation/random drift together determine the evolutionary fate of a binding site, as suggested by Mustonen and Lassig [19].

An unexpected result of our analyses is that the degree of homotypic clustering does not affect turnover rate. This is contrary to the notion that more binding sites of the same type will lead to greater redundancy, easing the selective pressure on the individual sites. Instead, the number of binding sites seems to be important to CRM function. This observation is similar to one of the implications of our findings of site-level selection: that exact affinities of binding sites are functionally important. Both observations are consistent with the so called “gradient threshold model” [47], which suggests that different genes may respond to different concentration levels of the same TF by harnessing different numbers of binding sites with varying affinities. The exact binding affinities and number of sites are important under this model. In a more detailed analysis of homotypic clustering, now considering the binding site arrangement, we observed that for some factors, if a site is adjacent to another site of the same factor, this site will be less likely to be lost during evolution. This may be indicative of cooperative activity of proximal homotypic binding sites, leading to stronger selective pressure. For instance, the significant result (p-value 0.0184, Table 6) for *Cad* is consistent with anecdotal evidence of *Cad* sites being located as proximal pairs [67]–[69], although we are not aware of any biochemical evidence for such cooperativity. There is also some evidence in the literature for DNA binding by homodimers of *Tll* [70] and *Hb* [71]. Our observation also suggests that sites that have a proximal “partner” are perhaps less likely to be spurious sites, which will provide a useful additional guideline to binding site prediction [72]. Surprisingly, we did not observe significant result for *Bcd*, even though it is known to bind cooperatively [52]. This negative result is a reminder that the sensitivity of our statistical tests may be reduced due to a variety of factors, e.g., alignment errors, false sites, etc. These factors are unlikely, however, to produce spurious statistical signals.

We found that the presence of a binding site for a different factor, either overlapping or proximal to a binding site, can strongly affect the latter's evolution. Different mechanisms of local interactions between sites are known in developmental CRMs, e.g., cooperative binding between two factors [73],[74], short-range quenching [75],[76], competitive binding to overlapping sites [74], etc. In all these cases, the loss of a single binding site may disrupt the interaction and create a larger change of expression than if the binding sites act in an additive fashion. As a consequence, these locally interacting site pairs may be under stronger selection. Our results support the importance of context in determining evolutionary fate of binding sites. A recent paper reports similar results for four CRMs of the *even-skipped* gene [53]. By working on a much larger set of CRMs, we confirm this context-dependence as a general evolutionary pattern. We also found some interesting specific cases, for example, the *Kr* sites that overlap with another TF site, appear more conserved, consistent with the known role of *Kr* as a repressor with the ability of competitive binding. In addition, the difference of the evolutionary patterns of the seven TFs suggests that they may depend on different mechanisms for their function. For example, both *Kr* and *Tll* are repressors, but *Tll* is more conserved if it is adjacent to some other site, while *Kr* is more conserved if it overlaps with another site. This seems to suggest that the relative importance of competitive binding and short-range quenching may be different in *Kr* and *Tll*.

We did not find strong evidence of suppression of large indels within CRMs relative to their flanking sequences. Our results are different from an earlier study of indel patterns of CRMs in sea urchins, which reports that large indels (>20 bp in length) are virtually absent inside CRM sequences [27]. There is an alternative explanation for this discrepancy: it has been known that *Drosophila* has a very compact genome as the neutral deletion rate is very high [77] and a large fraction (40–50% from different estimates) of intergenic non-coding sequences is under evolutionary constraint [48],[78]. Consequently, the flanking sequences of CRMs may not be entirely neutral, and the distinction between CRM and flanking sequences may not be as pronounced as in other species. (Our options were limited with respect to the “background” sequence to contrast with, since long repeats often used as neutral sequence in mammalian genomes [79] are rare in *Drosophila*.) The fact that short deletions are more constrained than short insertions is likely due to different effects of insertions and deletions on CRM sequences: any deletions that extend to an existing binding site will annul its functionality, while insertions, unless occurring exactly inside TFBSs, will only change the distance between sites, but not destroy them. In Text S1, we outline an illustrative calculation, suggesting that under simple but reasonable assumptions, short deletions are maybe twice as more likely to interfere with a binding site than are short insertions. These results combined with the lack of strong constraint on spacer sequences suggest that CRM structure is overall flexible, permits relatively quick evolutionary change, and functions without being very sensitive to the precise distances between binding sites. In terms of its implications for bioinformatics, our results seem to indicate that the indel signature can be a useful CRM predictor but not strong enough to work alone, somewhat contrary to prior expectations [27],[28].

## Methods

### Data

12 *Drosophila* genome sequences from *D. ananassae* (Feb. 2006 assembly), *D. erecta* (Feb. 2006 assembly), *D. grimshawi* (Feb. 2006 assembly), *D. melanogaster* (Apr. 2006 assembly, release 5), *D. mojavensis* (Feb. 2006 assembly), *D. persimilis* (Oct. 2005 assembly), *D. pseudoobscura* (Feb. 2006 assembly), *D. sechellia* (Oct. 2005 assembly), *D. simulans* (Apr. 2005 assembly), *D. virilis* (Feb. 2006 assembly), *D. willistoni* (Feb. 2006 assembly), and *D. simulans* (Nov. 2005 assembly) were compiled from UCSC Genome Browser database [80]. To predict the positions of putative TFBSs, position weight matrices (PWMs) for seven TFs, *Bcd* (*Bicoid*), *Cad* (*Caudal*), *Dstat*, *Hb* (*Hunchback*), *Kni* (*Knirps*), *Kr* (*Kruppel*), and *Tll* (*Tailless*) were compiled from FlyReg [81] and the literature. We used the phylogenetic tree and branch lengths for the 12 species in [82] and for the four species (*D. melanogaster*, *D. simulans*, *D. yakuba*, and *D. erecta*) in [25]. Orthologous sequences of each *D. melanogaster* CRM were obtained by the liftOver program from the UCSC Genome Browser database. The background region corresponding to a CRM was defined as the region upstream of the farthest known CRM of its target gene, equal in length to its corresponding CRM.

### Multiple Sequence Alignment and Insertion/Deletion Annotations

For the analysis of TFBS evolution, we developed a new multiple alignment program, “ProbconsMorph”, by integrating Probcons [41], a consistency based multiple sequence alignment program, and Morph [38], a pair-wise sequence alignment program that is specially designed to align regulatory modules. Morph uses a pair-HMM as a generative model for alignment of two orthologous CRMs, and is parameterized by the given motifs, as well as various evolutionary rate parameters that it fits to the data. It uses maximum likelihood inference to simultaneously perform TFBS annotation and alignment. It reports for every pair of positions in the two sequences, the posterior probability that they are aligned. Morph was run to produce such a probabilistic alignment of every pair of species. Probcons takes such pair-wise alignment probabilities and builds a multiple sequence alignment progressively, while using the “consistency transformation”: the probability of alignment of two nucleotides 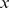 and 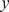 is updated based on the alignment probabilities of 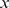 and 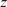 and of 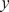 and 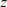, where 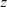 is a nucleotide from a third species. We have shown previously that Morph provides practical benefits for inference of evolutionary events and rates by computing a better alignment; ProbconsMorph is an effective and efficient extension of this program to more than two species. We made two simple modifications to Probcons to integrate it with Morph: firstly, Probcons was made to work on DNA sequences (the current implementation handles protein sequences only), and secondly, it was made to accept a phylogenetic tree as input, rather than estimate the tree at run-time. The ProbconsMorph software is publicly available at our site http://europa.cs.uiuc.edu/TFBSevolution/.

Pecan [42] was used for the alignment of four species in the analysis of indels in CRMs and spacers. We have performed extensive studies on simulated data to determine the limits of indel annotation, and estimated that accurate labeling of insertions and deletions is only possible for the four closely related species *D. melanogaster*, *D. simulans*, *D. yakuba*, and *D. erecta*. (Kim and Sinha, in preparation.) Pecan alignments of these four species, and *D. sechellia*, were also used for the study of position-specific substitution rates in binding sites (Table 1).

Insertion and deletion annotations were done using our previously published Indelign program [39] that is based on a probabilistic model of indels and annotates indels as being insertions or deletions based on maximum likelihood. We used a mixture of two geometric distributions as a model of the length distribution of indels. As shown in Figure S10, this mixture model is a much better fit to the indel length distributions empirically observed in *D. melanogaster* CRMs used in this study and their orthologous sequences in *D. simulans*, *D. yakuba*, and *D. erecta*. The new version of the Indelign program is available at our site http://europa.cs.uiuc.edu/TFBSevolution/.

### TFBS Analysis

#### Binding Site Annotation

The seven PWMs were used to scan *D. melanogaster* CRMs and their orthologous sequences for binding sites. For each substring of PWM-length, the log-likelihood ratio (*LLR*) scores for both orientations were computed. The LLR score with p-value 0.001 was used as the default threshold for prediction of binding sites. (The threshold was computed by an efficient recursive method in [13].) We used ChIP-based binding data [43] on *Bcd*, *Cad*, *Hb*, *Kr*, and *Kni* to limit the binding site annotation only to those CRMs that overlapped a ChIP-bound region (1% FDR) by at least 50%. We estimated the false positive proportion in our binding site predictions, in four different ways, as is described (with results) in Text S1. An eighth motif, *Giant*, that had been chosen for analysis, was discarded at this stage, due to very high estimated false positive proportions.

#### Binding Site Strength

The *LLR* score of a site is the logarithm of the ratio of (i) the likelihood of sampling the site from a PWM to (ii) the likelihood of sampling it from a background frequency distribution. TFBS strength was computed as the average of the *LLR* scores in the orthologous TFBS set.

#### Position-Specific Evolutionary Rates

For each orthologous TFBS set containing binding sites in all species, a parsimony cost for each position was computed. The average of this parsimony cost, over all orthologous TFBS sets, was used as the evolutionary rate of the position. For this analysis, orthologous TFBS sets were obtained differently: Pecan alignments of five closely related species (*D. melanogaster*, *D. sechellia*, *D. simulans*, *D. simulans*, *D. erecta*) were used, binding sites were predicted only in *D. melanogaster* and no PWM-based realignment was done in the other four species. This was done in order to avoid a bias in the analysis, and alignment errors were not a major concern since the species are very closely related.

#### Simulation of Binding Site Evolution

To simulate the evolution of a binding site, we repeat two steps: (i) compute the rate of each substitution event at each position according to HB model [12] or Equations (2) and (3) of SS model, and (ii) choose a substitution event with probability proportional to the rates of the event. The site is then updated according to this event, and the time is also incremented by an exponential random variable with mean equal to the inverse of the total rates of all events. The procedure is run until a pre-specified time (the divergence between two species studied) has been reached. We simulate a large number of sites to generate the histograms in Figure 2. For the mutation rates needed in HB and SS models, we use the HKY model and the parameters estimated from an earlier study [25]. The nucleotide frequencies in the background sequences are 0.3, 0.2, 0.2 and 0.3 for A, C, G, T respectively, and the transition-transversion bias equals to 2.0.

#### Comparison of Site-level Selection (SS) and Halpern-Bruno (HB) models

For each factor, the collection of sites was divided into two randomly chosen subsets: the first, called the training set, was used to learn a value of *4Ns* for the SS model, and the second, called the test set, was used to compare the two models. The histogram of energy difference values of sites in the test set was compared to a predicted histogram from either model (previous paragraph), using a “sum of squared errors” or SSE. The random split into training and test sets was repeated 100 times, and the SSE scores of each model were compared using a paired Wilcoxon signed rank test. The predicted histogram of a model was obtained as follows: a *D. melanogaster* binding site was chosen at random from the test set, and subjected to simulated evolution under the model, for the divergence time of *D. melanogaster* and *D. yakuba*, thus giving us a site pair, and its energy difference. This was then repeated 10^6^ times, to obtain a histogram of energy difference values. To obtain the SSE values shown in Figure 2 and Figure S4 and S5, we used the entire collection of sites as the training as well as test set.

#### Molecular Clock and Loss Rates

Let 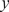 be the fraction of binding sites in *D. melanogaster* that have an ortholog in species 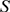 that is also a binding site, and let 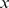 be the divergence time between the two species. We plotted 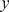 versus 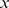 for different values of 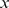 representing 11 different species, fitting to a line 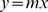. The negative slope, i.e., 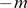 is called the “loss rate” in Table 4. Random motifs were obtained from the real motifs used in our analysis (see Methods). Sites for random PWMs were predicted and their loss rate calculated in the same way as for TFBSs. The mean and standard deviation of loss rate over 100 random PWMs is shown. The molecular clock test was repeated with a “correction” for false positive sites, as follows: If *F* is the false positive proportion, then in a collection of *n* predicted sites, *n-nF* are expected to be true sites, and if we observed *m* of the original *n* sites to be conserved, then an estimated *m-nFr* of these sites are true sites, where *r* is the proportion of “false” sites that are conserved (estimated from intronic regions not bound in ChIP assays). This would give us a “corrected” conservation probability as *(m−nFr)/(n−nF)*. We performed regression analysis of this corrected conservation probability (versus time), while simultaneously estimating a false positive proportion *F*, i.e., leaving *F* as a free parameter. Adjusted *R^2^* values were computed as *1−(n−1)(1−R^2^)/(n−k−1)* where *k+1* is the number of free parameters (2 in our case–the false positive proportion and the slope of the line; the intercept was fixed at 1.) The optimal values of *F* are reported in Table 3. (These estimates of false positive proportion range between 15% and 32%, depending on the motif.)

#### TFBS Turnover Rate

We first constructed a phylogenetic tree for each orthologous TFBS set by labeling a leaf node as “1” if its corresponding species has the site and 0 otherwise; a subtree rooted at the least common ancestor of leaf nodes labeled 1 was then identified. The turnover rate was defined as the parsimony cost calculated in the subtree, divided by the sum of branch lengths of the subtree. The overall TFBS turnover rate across multiple orthologous TFBS sets was defined as the sum of the parsimony costs of the individual orthologous TFBS sets, divided by the sum of branch lengths (obtained as described above) (see Figure S11 for the example of turnover rate calculation). The subtree should have at least 2 leaf nodes. We note that our definition of turnover rate is closely related to the Branch Length Score (BLS) used in [83]. When the least common ancestor is a binding site, then the inverse of our turnover rate is the expected time the site is conserved, i.e., the expected BLS.

#### Local Evolutionary Rates

The evolutionary rate of a CRM was defined as the sum of branch lengths estimated by Paml [50] with a given phylogenetic tree and the multiple alignment of the CRM. The test was done separately for each TF, and binding sites of that TF were “masked out” before estimating the CRM evolutionary rate. The ratio of the evolutionary rate in a CRM to that of introns in its neighboring gene was used as the local evolutionary rate.

#### Random PWMs

These were constructed by starting with one of the seven original PWMs, randomly permuting columns, and then randomly permuting rows for A and T, and rows for C and G, to obtain a random PWM that retains the information content and G/C content of the original.

#### Correlation Test

Bins were defined by the values of statistic (i.e., TFBS strength, rate of CRM, or distance between adjacent TFBSs). For the collection of samples in each bin, the overall TFBS turnover rate and the average of statistic were calculated, and the correlation test between them was performed.

## Supporting Information

Figure S1 An example of the graphic visualization of alignments of CRMs with binding site annotation.
(1.25 MB DOC)

Figure S2 Correlation between the specificity of a TFBS position and its evolutionary rate, with Pecan alignments.
(0.84 MB DOC)

Figure S3 Correlation between the specificity of a TFBS position and its evolutionary rate, with ProbconsMorph alignments.
(0.93 MB DOC)

Figure S4 Distributions of energy difference from observed binding sites (Observed), and those simulated by HB (HB) and Site-level Select (SS) models, with ProbconsMorph alignments.
(0.83 MB DOC)

Figure S5 Distributions of energy difference from observed binding sites (Observed), and those simulated by HB (HB) and Site-level Select (SS) models, with Pecan alignments.
(0.93 MB DOC)

Figure S6 Distributions of the number of substitutions from observed binding sites (Observed), and those simulated by HB (HB) and Site-level Selection (SS) models, with ProbconsMorph alignments.
(0.69 MB DOC)

Figure S7 Distributions of the number of substitutions from observed binding sites (Observed), and those simulated by HB (HB) and Site-level Selection (SS) models, with Pecan alignments.
(0.80 MB DOC)

Figure S8 The fraction of *D. melanogaster* TFBSs that are conserved in a related species (y-axis), as a function of the divergence time to that species (x-axis), with ProbconsMorph alignments.
(0.47 MB DOC)

Figure S9 The fraction of *D. melanogaster* TFBSs that are conserved in a related species (y-axis), as a function of the divergence time to that species (x-axis), with Pecan alignments.
(0.58 MB DOC)

Figure S10 Comparison of the two different indel length distributions, a single geometric distribution and a mixture of two geometric distributions.
(0.22 MB DOC)

Figure S11 An example of the calculation of TFBS turnover rate.
(0.05 MB DOC)

Table S1 Correlation between the specificity of a TFBS position and its evolutionary rate, with ProbconsMorph alignments.
(0.03 MB DOC)

Table S2 Comparison of HB and SS models, with Pecan alignments.
(0.03 MB DOC)

Table S3 Goodness-of-fit of a linear model for the fraction of conserved binding sites over divergence time, with Pecan alignments.
(0.03 MB DOC)

Table S4 Comparison of loss rates of binding sites using real and random motifs, with Pecan alignments.
(0.03 MB DOC)

Table S5 Correlation between TFBS strength and TFBS turnover rate, with Pecan alignments.
(0.03 MB DOC)

Table S6 Correlation between the distance between two adjacent homotypic sites and TFBS turnover rate, with Pecan alignments.
(0.03 MB DOC)

Table S7 Binding site conservation and its spatial context, with Pecan alignments.
(0.03 MB DOC)

Table S8 Correlation between evolutionary rate of CRM and TFBS turnover rate, with ProbconsMorph alignments.
(0.03 MB DOC)

Table S9 Correlation between evolutionary rate of CRM and TFBS turnover rate, with Pecan alignments.
(0.03 MB DOC)

Text S1 Supporting text.
(0.09 MB DOC)
