# Supplementary material for: Evolution of Regulatory Sequences in 12 Drosophila Species
Source: PLoS Genet. 2009 Jan 9;5(1):e1000330. doi: 10.1371/journal.pgen.1000330 (PMC2607023; doi:10.1371/journal.pgen.1000330)
Supplement: Figure S1 — An example of the graphic visualization of alignments of CRMs with binding site annotation. (1.25 MB DOC) [file pgen.1000330.s001.doc]

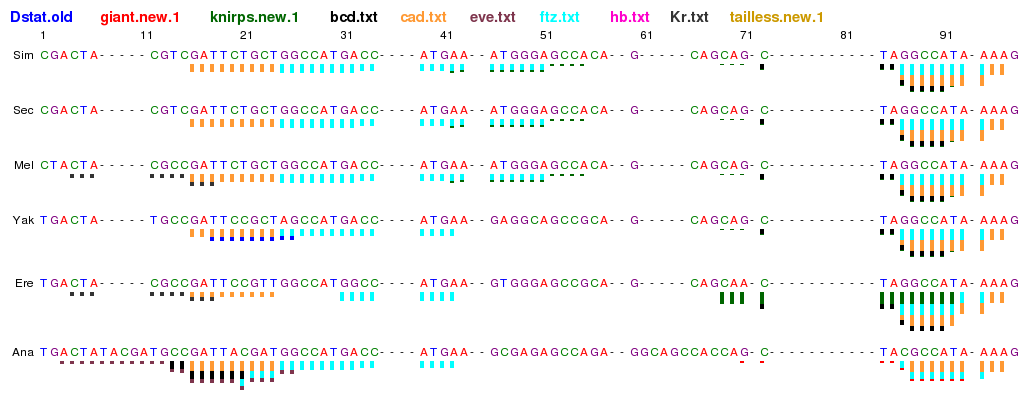


Figure S1. An example of the graphic visualization of alignments of CRMs with binding site annotation. The first row shows the names and colors of different TFs used in the visualization. The small rectangles below aligned sequences represent the positions of predicted TFBSs. The height of the rectangle means a relative log-likelihood ratio score. If there are multiple TFBSs at the same position, the rectangles for each TFBS are stacked together.
