# Supplementary material for: Evolution of Regulatory Sequences in 12 Drosophila Species
Source: PLoS Genet. 2009 Jan 9;5(1):e1000330. doi: 10.1371/journal.pgen.1000330 (PMC2607023; doi:10.1371/journal.pgen.1000330)
Supplement: Figure S3 — Correlation between the specificity of a TFBS position and its evolutionary rate, with ProbconsMorph alignments. (0.93 MB DOC) [file pgen.1000330.s003.doc]

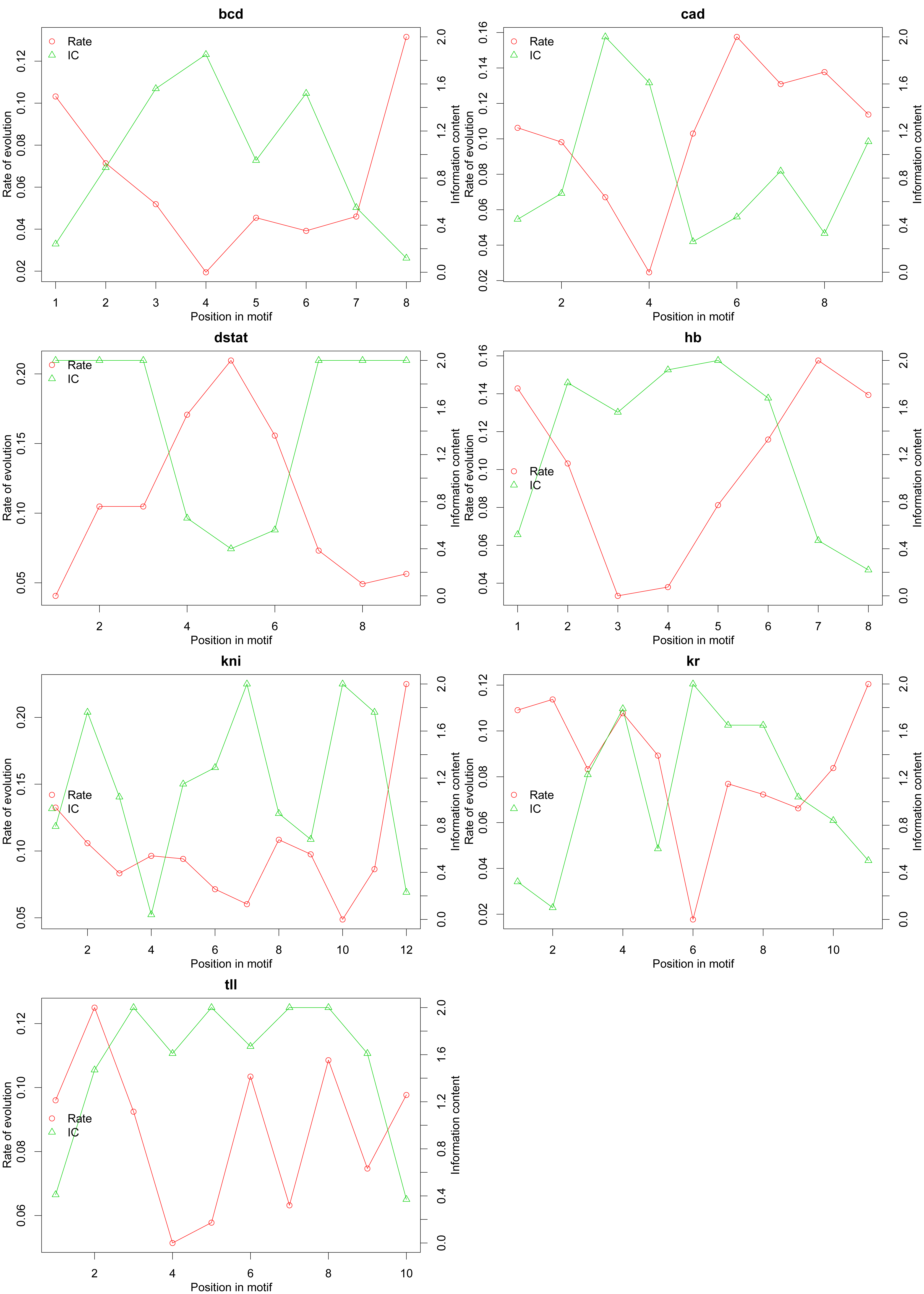


Figure S3. Correlation between the specificity of a TFBS position and its evolutionary rate, with ProbconsMorph alignments.
