# Supplementary material for: Evolution of Regulatory Sequences in 12 Drosophila Species
Source: PLoS Genet. 2009 Jan 9;5(1):e1000330. doi: 10.1371/journal.pgen.1000330 (PMC2607023; doi:10.1371/journal.pgen.1000330)
Supplement: Figure S4 — Distributions of energy difference from observed binding sites (Observed), and those simulated by HB (HB) and Site-level Select (SS) models, with ProbconsMorph alignments. (0.83 MB DOC) [file pgen.1000330.s004.doc]

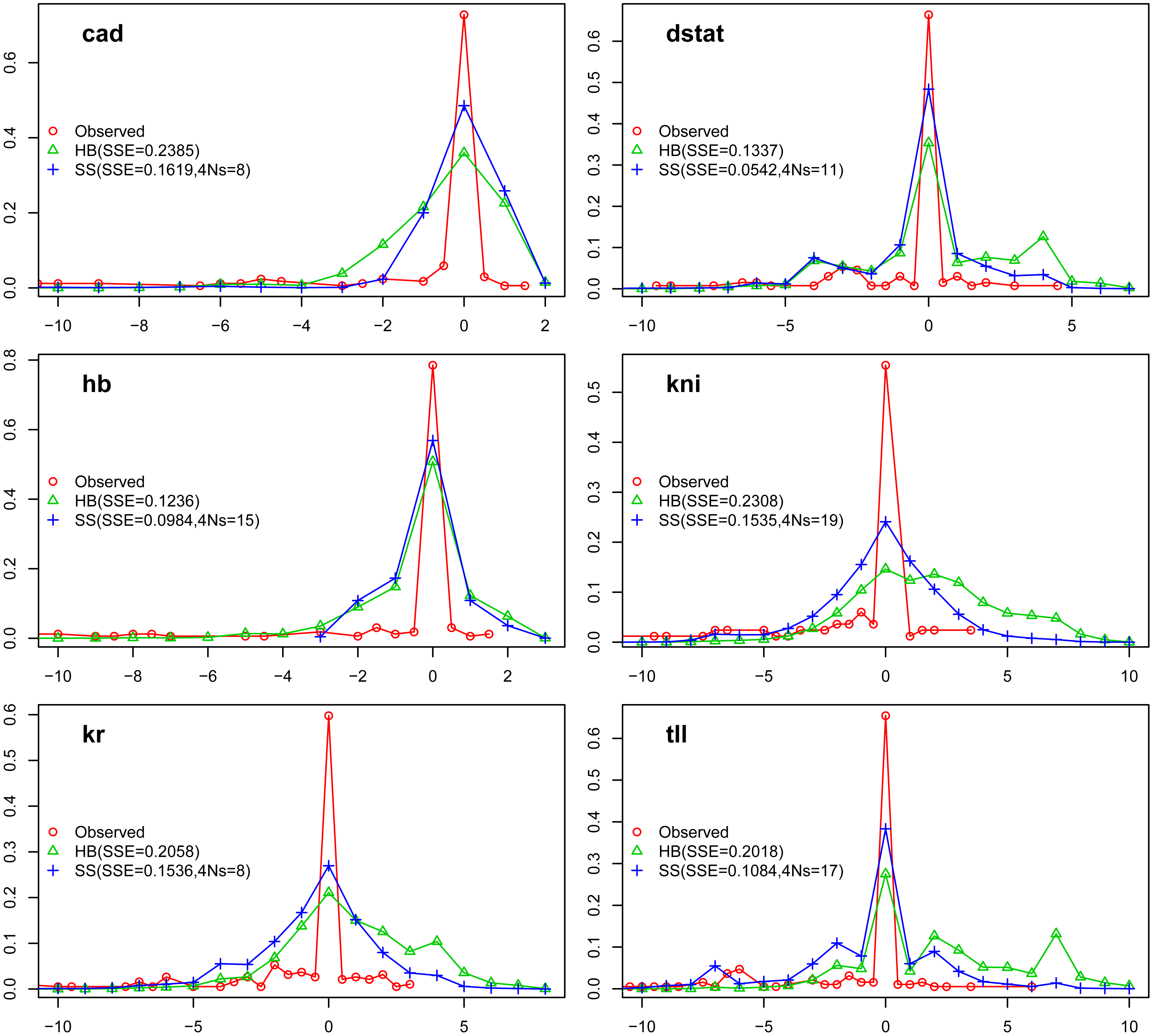


Figure S4. Distributions of energy difference calculated by subtracting the energy of an orthologous site in *D. yakuba* from that of a site in *D. melanogaster* from observed binding sites (Observed), and those simulated by HB (HB) and Site-level Selection (SS) models, with ProbconsMorph alignments. The x and y axes represent energy difference and the density of each difference respectively. SSE is the sum of squared errors between the observed and the simulated distributions and “*4Ns*” is the optimal value of the free parameter of the SS model.
