# Supplementary material for: Evolution of Regulatory Sequences in 12 Drosophila Species
Source: PLoS Genet. 2009 Jan 9;5(1):e1000330. doi: 10.1371/journal.pgen.1000330 (PMC2607023; doi:10.1371/journal.pgen.1000330)
Supplement: Figure S7 — Distributions of the number of substitutions from observed binding sites (Observed), and those simulated by HB (HB) and Site-level Selection (SS) models, with Pecan alignments. (0.80 MB DOC) [file pgen.1000330.s007.doc]

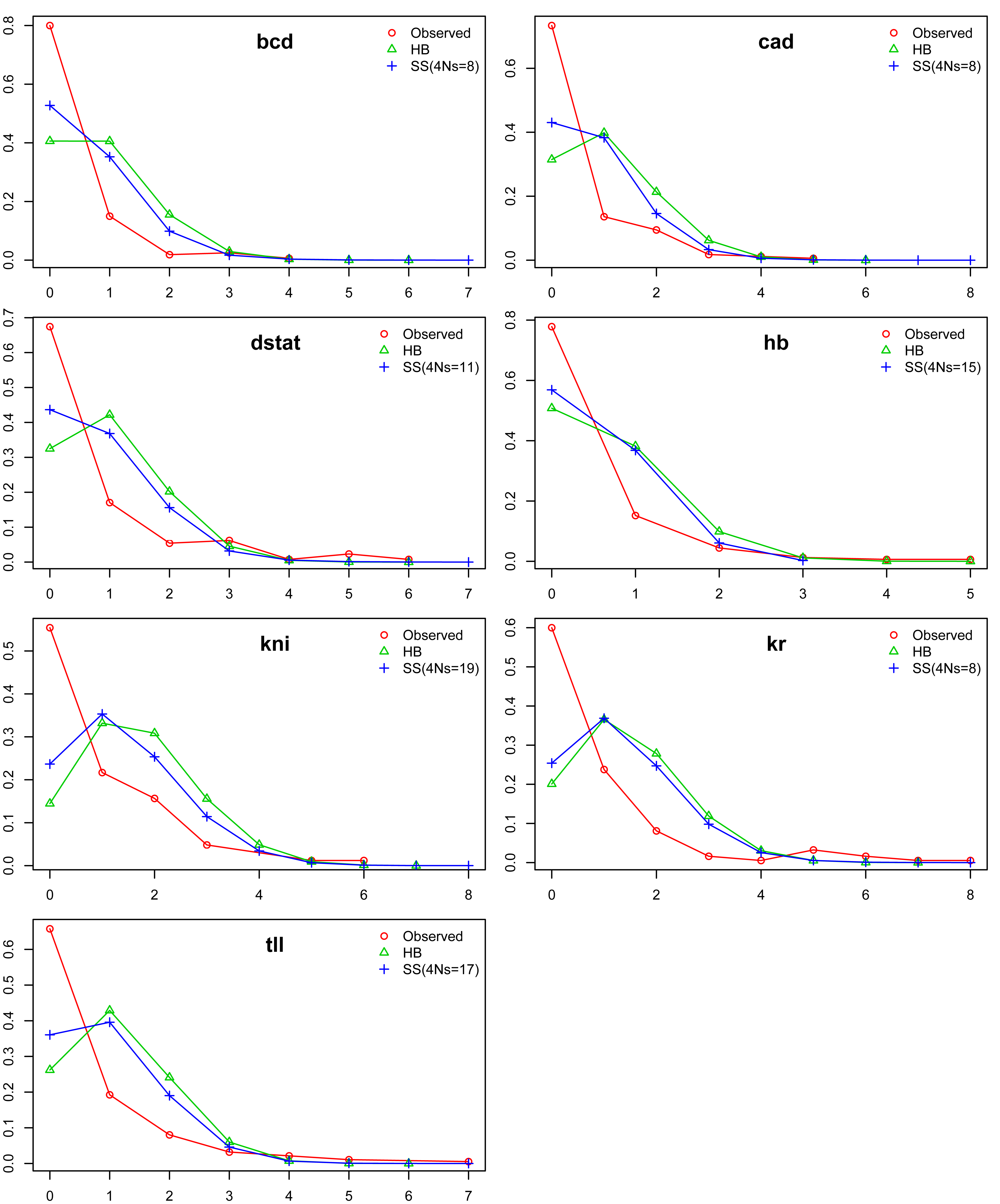


Figure S7. Distributions of the number of substitutions between *D. melanogaster* and *D. yakuba* sites from observed binding sites (Observed), and those simulated by HB (HB) and Site-level Selection (SS) models, with Pecan alignments. The x and y axes represent the number of substitutions and the density of each number respectively. “*4Ns*” is the optimal value of the free parameter of the SS model.
