# Supplementary material for: Evolution of Regulatory Sequences in 12 Drosophila Species
Source: PLoS Genet. 2009 Jan 9;5(1):e1000330. doi: 10.1371/journal.pgen.1000330 (PMC2607023; doi:10.1371/journal.pgen.1000330)
Supplement: Figure S8 — The fraction of D. melanogaster TFBSs that are conserved in a related species (y-axis), as a function of the divergence time to that species (x-axis), with ProbconsMorph alignments. (0.47 MB DOC) [file pgen.1000330.s008.doc]

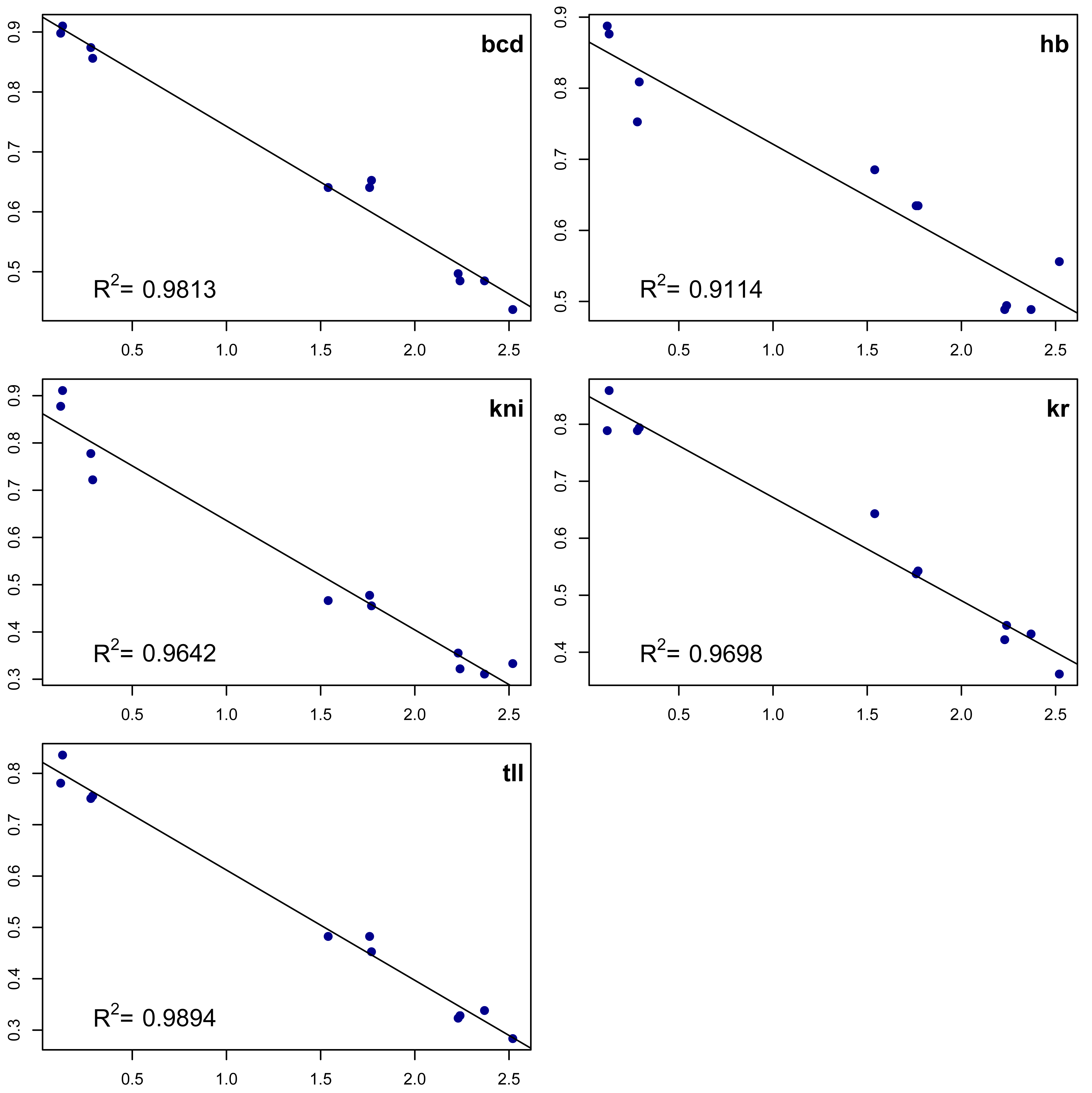


Figure S8. The fraction of *D. melanogaster* TFBSs that are conserved in a related species (y-axis), as a function of the divergence time to that species (x-axis), with ProbconsMorph alignments.
