# Supplementary material for: Evolution of Regulatory Sequences in 12 Drosophila Species
Source: PLoS Genet. 2009 Jan 9;5(1):e1000330. doi: 10.1371/journal.pgen.1000330 (PMC2607023; doi:10.1371/journal.pgen.1000330)
Supplement: Figure S10 — Comparison of the two different indel length distributions, a single geometric distribution and a mixture of two geometric distributions. (0.22 MB DOC) [file pgen.1000330.s010.doc]

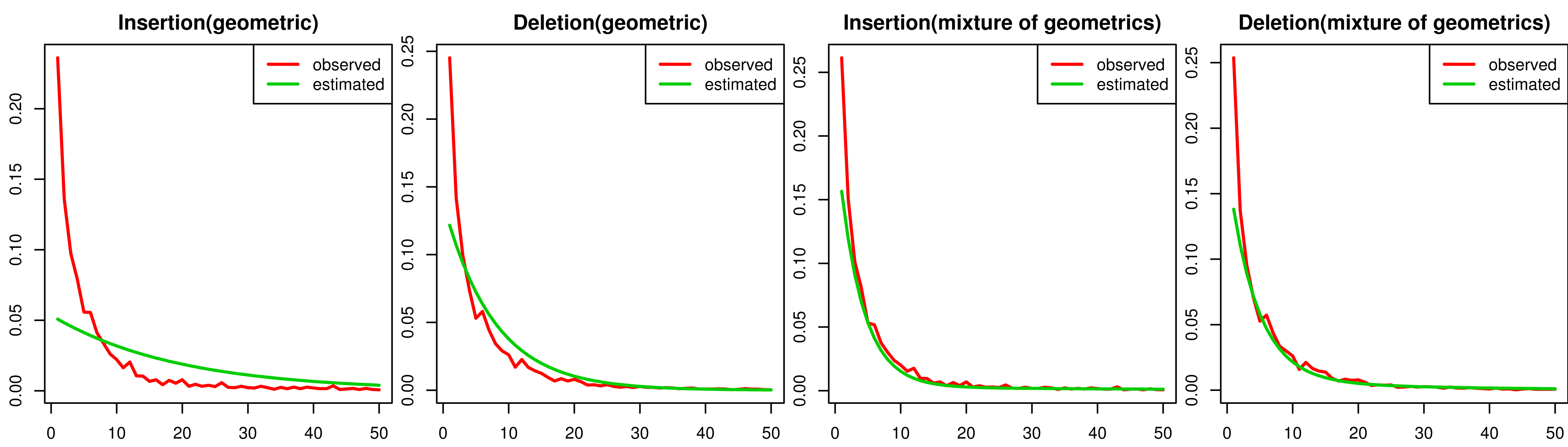


Figure S10. Comparison of the two different indel length distributions, a single geometric distribution and a mixture of two geometric distributions. The x and y axes represent the length of indels and the density of each length respectively.
