# Supplementary material for: Evolution of Regulatory Sequences in 12 Drosophila Species
Source: PLoS Genet. 2009 Jan 9;5(1):e1000330. doi: 10.1371/journal.pgen.1000330 (PMC2607023; doi:10.1371/journal.pgen.1000330)
Supplement: Figure S11 — An example of the calculation of TFBS turnover rate. (0.05 MB DOC) [file pgen.1000330.s011.doc]

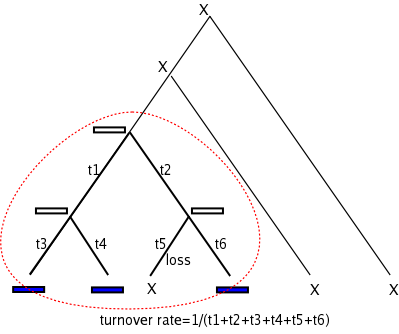


Figure S11. An example of the calculation of TFBS turnover rate. Among 6 species, only 3 species have a binding site (rectangles at the leaves). The subtree rooted at the least common ancestor of the binding sites is identified (in the dashed circle). There is one loss event in the subtree and, thus, the turnover rate is 1 (the number of events) divided by the sum of t1 though t6 (branch lengths in the subtree).
