# Supplementary material for: Evolution of Regulatory Sequences in 12 Drosophila Species
Source: PLoS Genet. 2009 Jan 9;5(1):e1000330. doi: 10.1371/journal.pgen.1000330 (PMC2607023; doi:10.1371/journal.pgen.1000330)
Supplement: Table S1 — Correlation between the specificity of a TFBS position and its evolutionary rate, with ProbconsMorph alignments. (0.03 MB DOC) [file pgen.1000330.s012.doc]

Table S1. Correlation between the specificity of a TFBS position and its evolutionary rate, with ProbconsMorph alignments

| Factor | Number of TFBSs | Width of motif | Correlation coefficient^a^ | P-value |
| --- | --- | --- | --- | --- |
| bcd | 160 | 8 | -0.81 | **0.0109** |
| cad | 174 | 9 | -0.48 | 0.0969 |
| dstat | 129 | 9 | -0.83 | **0.0031** |
| hb | 175 | 8 | -0.67 | **0.0415** |
| kni | 86 | 12 | -0.71 | **0.0052** |
| kr | 179 | 11 | -0.72 | **0.0058** |
| tll | 187 | 10 | -0.27 | 0.2255 |

^a^Spearman’s correlation coefficient.
