# Supplementary material for: Evolution of Regulatory Sequences in 12 Drosophila Species
Source: PLoS Genet. 2009 Jan 9;5(1):e1000330. doi: 10.1371/journal.pgen.1000330 (PMC2607023; doi:10.1371/journal.pgen.1000330)
Supplement: Table S2 — Comparison of HB and SS models, with Pecan alignments. (0.03 MB DOC) [file pgen.1000330.s013.doc]

Table S2. Comparison of HB and SS models, with Pecan alignments

| Factor | Median SSE^a^ | | P-value^b^ | *4Ns*^c^ |
| --- | --- | --- | --- | --- |
|  | HB model | SS model |  |  |
| bcd | 0.18 | 0.10 | <2.20E-16 | 8 |
| cad | 0.23 | 0.16 | <2.20E-16 | 8 |
| dstat | 0.13 | 0.06 | <2.20E-16 | 11 |
| hb | 0.09 | 0.07 | <2.20E-16 | 15 |
| kni | 0.21 | 0.16 | <2.20E-16 | 19 |
| kr | 0.19 | 0.15 | <2.20E-16 | 8 |
| tll | 0.18 | 0.10 | <2.20E-16 | 17 |

^a^Median values of sum of squared errors (SSE) from 100 different simulations with the model.

^b^P-value from paired Wilcoxon signed-rank test.

^c^Optimal value of the free parameter of SS model.
