# Supplementary material for: Evolution of Regulatory Sequences in 12 Drosophila Species
Source: PLoS Genet. 2009 Jan 9;5(1):e1000330. doi: 10.1371/journal.pgen.1000330 (PMC2607023; doi:10.1371/journal.pgen.1000330)
Supplement: Table S3 — Goodness-of-fit of a linear model for the fraction of conserved binding sites over divergence time, with Pecan alignments. (0.03 MB DOC) [file pgen.1000330.s014.doc]

Table S3. Goodness-of-fit of a linear model for the fraction of conserved binding sites over divergence time, with Pecan alignments

| Factor | R^2^ (raw data)^a^ | Adjusted R^2^ (corrected data)^b^ | FP^c^ |
| --- | --- | --- | --- |
| bcd | 0.9695 | 0.9490 | 0.12 |
| cad | 0.9846 | 0.9688 | 0.30 |
| dstat | 0.9958 | 0.9835 | 0.26 |
| hb | 0.9674 | 0.9636 | 0.23 |
| kni | 0.9565 | 0.9814 | 0.31 |
| kr | 0.9827 | 0.9261 | 0.28 |
| tll | 0.9870 | 0.9550 | 0.33 |

^a^R^2^ from raw data without correcting for the false positive rate.

^b^Adjusted R^2^ from data corrected for the false positive rate.

^c^Estimated false positive rate obtained by regression.
