# Supplementary material for: Evolution of Regulatory Sequences in 12 Drosophila Species
Source: PLoS Genet. 2009 Jan 9;5(1):e1000330. doi: 10.1371/journal.pgen.1000330 (PMC2607023; doi:10.1371/journal.pgen.1000330)
Supplement: Table S4 — Comparison of loss rates of binding sites using real and random motifs, with Pecan alignments. (0.03 MB DOC) [file pgen.1000330.s015.doc]

Table S4. Comparison of loss rates of binding sites using real and random motifs, with Pecan alignments

|  |  | Random PWMs | |
| --- | --- | --- | --- |
| Factor | Loss rate | Mean | Stdev |
| bcd | 0.1909 | 0.2541 | 0.0222 |
| cad | 0.2009 | 0.2451 | 0.0214 |
| dstat | 0.2674 | 0.2649 | 0.0171 |
| hb | 0.1488 | 0.1944 | 0.0226 |
| kni | 0.2280 | 0.2564 | 0.0173 |
| kr | 0.1825 | 0.2679 | 0.0176 |
| tll | 0.2092 | 0.2401 | 0.0196 |

These rates are without false positive correction.
