# Supplementary material for: Evolution of Regulatory Sequences in 12 Drosophila Species
Source: PLoS Genet. 2009 Jan 9;5(1):e1000330. doi: 10.1371/journal.pgen.1000330 (PMC2607023; doi:10.1371/journal.pgen.1000330)
Supplement: Table S5 — Correlation between TFBS strength and TFBS turnover rate, with Pecan alignments. (0.03 MB DOC) [file pgen.1000330.s016.doc]

Table S5. Correlation between TFBS strength and TFBS turnover rate, with Pecan alignments

| Factor | Number of TFBS sets | Correlation coefficient^a^ | P-value | Random PWM^b^ |
| --- | --- | --- | --- | --- |
| bcd | 162 | -0.59 | **0.0035** | 0 |
| cad | 172 | -0.34 | 0.0696 | 15 |
| dstat | 128 | -0.46 | **0.0210** | 11 |
| hb | 160 | -0.53 | **0.0058** | 0 |
| kni | 84 | -0.44 | **0.0232** | 14 |
| kr | 186 | -0.66 | **0.0007** | 3 |
| tll | 187 | -0.86 | **<2.20E-16** | 0 |

^a^Spearman’s correlation coefficient.

^b^Number of random PWMs (out of 100 simulations) that show greater correlation than the real motif.
