# Supplementary material for: Evolution of Regulatory Sequences in 12 Drosophila Species
Source: PLoS Genet. 2009 Jan 9;5(1):e1000330. doi: 10.1371/journal.pgen.1000330 (PMC2607023; doi:10.1371/journal.pgen.1000330)
Supplement: Table S6 — Correlation between the distance between two adjacent homotypic sites and TFBS turnover rate, with Pecan alignments. (0.03 MB DOC) [file pgen.1000330.s017.doc]

Table S6. Correlation between the distance between two adjacent homotypic sites and TFBS turnover rate, with Pecan alignments

| Factor | Number of TFBSs | Correlation coefficient^a^ | P-value |
| --- | --- | --- | --- |
| bcd | 156 | 9.01E-04 | 0.4979 |
| cad | 166 | 0.19 | 0.1543 |
| dstat | 111 | 0.09 | 0.3129 |
| hb | 147 | 0.29 | **0.0490** |
| kni | 80 | 0.11 | 0.3074 |
| kr | 179 | 0.12 | 0.2538 |
| tll | 178 | 0.35 | **0.0236** |

^a^Spearman’s correlation coefficient.
