# Supplementary material for: Evolution of Regulatory Sequences in 12 Drosophila Species
Source: PLoS Genet. 2009 Jan 9;5(1):e1000330. doi: 10.1371/journal.pgen.1000330 (PMC2607023; doi:10.1371/journal.pgen.1000330)
Supplement: Table S7 — Binding site conservation and its spatial context, with Pecan alignments. (0.03 MB DOC) [file pgen.1000330.s018.doc]

Table S7. Binding site conservation and its spatial context, with Pecan alignments

| Factor | P vs D^a^ | O vs NO^b^ |
| --- | --- | --- |
| bcd | 0.9985^*^ | 0.3636 |
| cad | 0.5536 | **0.0045** |
| dstat | **0.0039** | 0.8148 |
| hb | 0.6024 | **0.0415** |
| kni | 0.5847 | 0.2539 |
| kr | 0.7012 | 0.0557 |
| tll | **0.0305** | 0.0861 |

Numbers are P-values by hypergeometric test.

^a^P means proximal and D means distal.

^b^O means overlap and NO means non-overlap.

*The opposite p-value is 0.01.
