# Supplementary material for: Evolution of Regulatory Sequences in 12 Drosophila Species
Source: PLoS Genet. 2009 Jan 9;5(1):e1000330. doi: 10.1371/journal.pgen.1000330 (PMC2607023; doi:10.1371/journal.pgen.1000330)
Supplement: Table S8 — Correlation between evolutionary rate of CRM and TFBS turnover rate, with ProbconsMorph alignments. (0.03 MB DOC) [file pgen.1000330.s019.doc]

Table S8. Correlation between evolutionary rate of CRM and TFBS turnover rate, with ProbconsMorph alignments

| Factor | Number of TFBS sets | Correlation coefficient^a^ | P-value |
| --- | --- | --- | --- |
| bcd | 113 | 0.06 | 0.4078 |
| cad | 133 | 0.29 | 0.0930 |
| dstat | 93 | 0.49 | **0.0127** |
| hb | 145 | 0.26 | 0.1302 |
| kni | 68 | 0.12 | 0.3178 |
| kr | 129 | 0.11 | 0.3141 |
| tll | 116 | 0.33 | 0.0745 |

^a^Spearman’s correlation coefficient.
