# Supplementary material for: Evolution of Regulatory Sequences in 12 Drosophila Species
Source: PLoS Genet. 2009 Jan 9;5(1):e1000330. doi: 10.1371/journal.pgen.1000330 (PMC2607023; doi:10.1371/journal.pgen.1000330)
Supplement: Table S9 — Correlation between evolutionary rate of CRM and TFBS turnover rate, with Pecan alignments. (0.03 MB DOC) [file pgen.1000330.s020.doc]

Table S9. Correlation between evolutionary rate of CRM and TFBS turnover rate, with Pecan alignments

| Factor | Number of TFBS sets | Correlation coefficient^a^ | P-value |
| --- | --- | --- | --- |
| bcd | 119 | -0.03 | 0.5357 |
| cad | 142 | 0.38 | 0.0648 |
| dstat | 99 | 0.35 | 0.0947 |
| hb | 153 | 0.08 | 0.3774 |
| kni | 66 | 0.20 | 0.2389 |
| kr | 132 | 0.02 | 0.4694 |
| tll | 128 | 0.38 | 0.0647 |

^a^Spearman’s correlation coefficient.
