# Supplementary material for: Evolution of Regulatory Sequences in 12 Drosophila Species
Source: PLoS Genet. 2009 Jan 9;5(1):e1000330. doi: 10.1371/journal.pgen.1000330 (PMC2607023; doi:10.1371/journal.pgen.1000330)
Supplement: Text S1 — Supporting text. (0.09 MB DOC) [file pgen.1000330.s021.doc]

**Short deletions are more likely to interfere with binding sites than are short insertions**

Consider a binding site of length $l$ in a CRM of length $L$. Consider an insertion or deletion of length $k$ bp. The insertion will “delete” the binding site if it is located at any of the $l-1$ positions within the site – an event of probability$l-1/L$. The deletion, on the other hand, will remove the site as long as its starting position is within the site *or less than* $k$ *bp away from the site* – an event of probability$(l+k-1)/L$. Now, assuming that the length distribution of insertions (resp., deletions) is given by $p_{I}(k)$ (resp., $p_{D}(k)$), we find that the probability of an insertion interfering the site is $\sum_{k} p_{I}(k)(l-1)/L$ and the corresponding probability for a deletion is $\sum_{k} p_{D}(k)(l+k-1)/L$. The difference between these two probabilities comes out to be , where $\mu_{D}$ is the average length of a deletion. Comparing this to the above-mentioned probability of an insertion interfering with the site, which is $\sum_{k} p_{I}(k)(l-1)/L\approx l/L$, we find that the ratio between the site interference probabilities for a deletion and an insertion is $\approx1+\mu_{D}/l$. Noting that the average deletion length is of the order of ~10*bp*, the same as a typical binding site’s length, we find that deletions are about twice as likely to interfere with a binding site.

**False positive proportion estimation**

**Method 1:** We estimated the false positive prediction rate for each transcription factor by considering only CRMs that overlap ChIP-bound regions for that TF (at 1% FDR, Li et al. 2008) and contrasting the density of predicted binding sites to that in intronic regions (excluding first introns) that do not overlap ChIP-bound regions(Table S10). The typical false positive proportions are ~ 35-50%, except for *Hb* and *Tll*, where it is upward of 60%. (Note: *Tll* estimates were done without using ChIP data, which was not available for this factor.) Note that this approach estimates only an upper bound on the false positive proportion, since some of the predicted sites in intronic regions may be true binding sites, which do not show binding in the conditions of the ChIP assay.

Table S10. False positive proportion based on contrast with “unbound” intronic sequences

| Factor | Number of CRMs | Number of sites in CRMs | Number of sites in introns | False positive rate |
| --- | --- | --- | --- | --- |
| bcd | 47 | 176 | 79 | 0.45 |
| cad | 51 | 189 | 65 | 0.35 |
| dstat | 68 | 139 | 54 | 0.39 |
| hb | 53 | 182 | 117 | 0.64 |
| kni | 34 | 94 | 47 | 0.51 |
| kr | 60 | 235 | 113 | 0.48 |
| tll | 68 | 213 | 137 | 0.65 |

**Method 2:** We adopted another approach to estimation of the false positive proportions, by performing evolutionary comparisons. Let *n* be the number of predicted sites in CRMs and *m* be the number of these that are evolutionarily conserved (by some criteria). Let the corresponding numbers in background regions (unbound intronic) be *n_0_* and *m_0_*. Let F be the false positive proportion, and let us assume that every true binding site is conserved. Since roughly *nF* false predictions are expected, and their approximate probability of conservation is *m_0_/n_0_*, we have the expectation that

$$\frac{m_{0}}{n_{0}}F+1\times(1-F)=\frac{m}{n}$$

Here, the observed conservation probability is formulated as a weighted average of the conservation probabilities of a false site and a true site. This allows us to estimate *F,* the false positive proportion. Note that if we relax the assumption that every true binding site is conserved, then the estimated *F* will be smaller, meaning that the above equation gives us overestimates of *F*.

The results of our estimates using this method are given in Table S11. (A for ProbconsMorph alignments, B for Pecan alignments). The estimated false positive proportion is in the range 29-45%.

Table S11A. False positive rate based on evolutionary comparisons, with ProbconsMorph alignments

| Factor | N^a^ | M^b^ | N_0_^c^ | M_0_^d^ | False positive rate |
| --- | --- | --- | --- | --- | --- |
| bcd | 163 | 125 | 2040 | 390 | 0.29 |
| cad | 168 | 105 | 1223 | 146 | 0.43 |
| dstat | 129 | 83 | 629 | 85 | 0.41 |
| hb | 168 | 120 | 2288 | 180 | 0.31 |
| kni | 86 | 55 | 1356 | 196 | 0.42 |
| kr | 191 | 122 | 1761 | 227 | 0.41 |
| tll | 188 | 126 | 1701 | 232 | 0.38 |

^a^Number of predicted sites in CRMs.

^b^Number of predicted sites that are evolutionarily conserved.

^c^Number of predicted sites in background regions.

^d^Number of predicted sites that are evolutionarily conserved in background regions.

Table S11B. False positive rate based on evolutionary comparisons, with Pecan alignments

| Factor | N^a^ | M^b^ | N_0_^c^ | M_0_^d^ | False positive rate |
| --- | --- | --- | --- | --- | --- |
| bcd | 162 | 125 | 2040 | 390 | 0.28 |
| cad | 172 | 104 | 1223 | 146 | 0.45 |
| dstat | 128 | 82 | 629 | 85 | 0.42 |
| hb | 160 | 116 | 2288 | 180 | 0.30 |
| kni | 84 | 55 | 1356 | 196 | 0.40 |
| kr | 186 | 120 | 1761 | 227 | 0.41 |
| tll | 187 | 125 | 1701 | 232 | 0.38 |

^a^Number of predicted sites in CRMs.

^b^Number of predicted sites that are evolutionarily conserved.

^c^Number of predicted sites in background regions.

^d^Number of predicted sites that are evolutionarily conserved in background regions.

**Method 3:** We used known (footprinted) binding sites for *Kr* from 7 CRMs to estimate the false positive proportion for this motif (obtained from baterial 1-hybrid data and not from the footprinted sites themselves). We found that 15 of 26 predicted sites in these CRMs were experimentally known, for a false positive proportion of 42%. Again, this is an overestimate, since some of the remaining 11 sites may be functional but were not footprinted.
